# Comparative Analysis of the Gut Microbial Communities of the Eurasian Kestrel (*Falco tinnunculus*) at Different Developmental Stages

**DOI:** 10.3389/fmicb.2020.592539

**Published:** 2020-12-18

**Authors:** Lei Zhou, Xiaona Huo, Boyu Liu, Hui Wu, Jiang Feng

**Affiliations:** ^1^College of Animal Science and Technology, College of Veterinary Medicine, Jilin Agricultural University, Changchun, China; ^2^School of Life Sciences, Jilin Agricultural University, Changchun, China

**Keywords:** *Falco tinnunculus*, gut microbiota, fecal sample, community composition, 16S rRNA high-throughput sequencing

## Abstract

The gut microflora play a very important role in the life of animals. Although an increasing number of studies have investigated the gut microbiota of birds in recent years, there is a lack of research work on the gut microbiota of wild birds, especially carnivorous raptors, which are thought to be pathogen vectors. There are also a lack of studies focused on the dynamics of the gut microbiota during development in raptors. In this study, 16S rRNA gene amplicon high-throughput sequencing was used to analyze the gut microbiota community composition of a medium-sized raptor, the Eurasian Kestrel (*Falco tinnunculus*), and to reveal stage-specific signatures in the gut microbiota of nestlings during the pre-fledging period. Moreover, differences in the gut microbiota between adults and nestlings in the same habitat were explored. The results indicated that the Eurasian Kestrel hosts a diverse assemblage of gut microbiota. Proteobacteria, Firmicutes, Actinobacteria, and Bacteroidetes were the primary phyla shared within the guts of adults and chicks. However, adults harbored higher abundances of Proteobacteria while nestlings exhibited higher abundances of Firmicutes and Actinobacteria, and consequently the majority of dominant genera observed in chicks differed from those in adults. Although no significant differences in diversity were observed across the age groups during nestling ontogeny, chicks from all growth stages harbored richer and more diverse bacterial communities than adults. In contrast, the differences in gut microbial communities between adults and younger nestlings were more pronounced. The gut microbes of the nestlings in the last growth stage were converged with those of the adults. This study provides basic reference data for investigations of the gut microbiota community structure of wild birds and deepens our understanding of the dynamics of the gut microflora during raptor development.

## Introduction

In animals, microorganisms occur not only externally, such as on skin and feathers, but also internally in the gastrointestinal and reproductive tracts of their hosts (McFall-Ngai et al., 2013; Colston and Jackson, 2016). Many studies have shown that gut microbiota play important roles in several fundamental and crucial physiological process in humans and other animals, such as development (Malmuthuge et al., 2015), immune homeostasis (Ahern et al., 2014), nutrient assimilation (Kau et al., 2011), and disease resistance (Kinross et al., 2011; Lee and Hase, 2014). Given the potentially profound impact of gut microbiota on host conditions, investigations of gut microbial assemblages within hosts are of high ecological and evolutionary importance (Ezenwa et al., 2012). In recent years, in order to better understand the interactions between gut microbes and their hosts, studies of gut microbes conducted on single-species or multi-species host have been carried out in many animal groups including mammals (Lu et al., 2012; Russell et al., 2012; Tung et al., 2015; Davis M. Y. et al., 2016; de Goffau et al., 2019; Xiao et al., 2019), birds (Ding et al., 2016, 2020; Grond et al., 2017), reptiles (Ren et al., 2016; Kohl et al., 2017), and fish (Davis D. J. et al., 2016). Studies on avian gut microbiota have increased rapidly since 2014 (Hird et al., 2015; Kohl et al., 2018; Wang et al., 2019b; Capunitan et al., 2020; Cho and Lee, 2020; Song et al., 2020), remain heavily outnumbered by studies of mammals, and are dominated by research on domestic poultry (Cui et al., 2017) or captive-bred model species (Ding et al., 2020). Therefore, wild birds remain understudied despite their relevance for pathogen transmission and for understanding diet and environmental influences on gut microbial structure and function.

The gastrointestinal tract is largely sterile in newborn vertebrates (Koenig et al., 2011; Grond et al., 2017) and is subsequently colonized by diverse bacterial taxa that vary in abundance and functional traits (Ley et al., 2008; Lozupone et al., 2012). One of the central questions in the study of host-associated microbial communities is how they are acquired and how the community structures vary with age. The developmental succession of the gut microbiota can have large implications for the biology of the host. For example, in some animals, perturbation of the gut microbiota early in life can alter adiposity and immune function and susceptibility to disease later in life (Russell et al., 2012; Knutie et al., 2017). Therefore, understanding of host age-related dynamics of gut microbial communities is essential for predicting their future states. However, information pertaining to the development of these gut microbiota communities during the period of nestling growth in natural populations of wild birds is extremely limited due to difficulties in sampling. Indeed, such studies have been conducted only in a few taxa such as seabirds (Barbosa et al., 2016), shorebirds (Grond et al., 2017), and passerines (Kohl et al., 2018). Carnivorous birds of prey have received less attention. Further, no studies have evaluated the age-related establishment and succession of gut microbiota in raptor nestlings during development to our knowledge. Moreover, most previous studies have sampled microbiome communities in chicks only once, just before fledging (Zheng et al., 2018). It is expected that nestlings are colonized by more species of microbes as they age and that the development of gut microbiota communities occurs gradually after hatching (Barbosa et al., 2016; Kreisinger et al., 2017; Wang et al., 2019a). However, some studies have shown that the gut microbiota diversity was not increased linearly as previously suspected; it was relatively stable (short-tailed shearwaters in Dewar et al., 2017) or strongly fluctuating (Teyssier et al., 2018) throughout host development. Moreover, there is no consensus on differences in intestinal microbial communities between nestlings and adults. In short, current evidence for how gut microbial communities develop is still rather puzzling, more studies are needed to explore developmental dynamics of gut microbial communities in altricial bird species with a longer pre-fledging period. Such investigations will help us to understand the diversity and dynamics of the gut microbiota communities in wild birds.

In this study, we investigated a medium-sized raptor, the Eurasian Kestrel (*Falco tinnunculus*), which is thought to be a potential pathogen vector and exhibits a unique diet and lifestyle. Firstly, *F. tinnunculus* mainly prey on small rodents, which carry a large number of pathogens. Additionally, *F. tinnunculus* does not build nests by themselves; they usually live in cliffs, tree holes, walls of old buildings, or reused old nests of magpies, crows, and other birds (wherein all the nests are already made); and they prefer to use artificial nesting boxes repeatedly in our study region. In these artificial nesting boxes, the bird’s feces and pellets cannot be discharged from the nest, which results in poor sanitation conditions in the nests, especially over long breeding periods, since pre-fledging periods last at least 30 days. Finally, *F. tinnunculus* have been reported to be an important carrier and infectious source of a variety of pathogenic microorganisms, even though these pathogens do not make them sick (Dipineto et al., 2015; Reuter et al., 2016; Pankovics et al., 2017). In this study, 16S rRNA gene amplicon high-throughput sequencing was used to (1) test the gut microbiota community composition characteristics of this species, (2) explore changes in the gut microbiota structure that occur during the relatively long pre-fledging period of nestlings, (3) and compare the gut microbiota community structure of nestlings at different growth stages with that of adults. The significance of this research is twofold. Firstly, the results presented herein enhance our understanding of the gut microflora of wild birds and the variations that occur in the gut microbiota during development in raptors. Secondly, our results laid an important foundation for our subsequent research on the prevalence of enteropathogens and the consequences of particular communities of bacteria for host health.

## Materials and Methods

### Sample Collection

We conducted this study in the Zuojia Natural Reserve in Jilin Province (126°1′–127°2′N, 44°6′–45°5′E) during the breeding season (from late March to late July) in 2019. We collected feces samples of *F. tinnunculus* using old artificial nesting boxes. The internal dimensions of the boxes were all the same: 50 cm deep with a 35 cm × 35 cm bottom area and a 12.5-cm- or 15-cm-diameter entrance hole near the top. The time at which the first nestling was hatched was recorded as the first day of the nesting period. During the nestling period, feces samples were collected every 5 days until all nestlings fledged. We adopted a non-invasive sampling method in which the nestlings were taken out of the nest box and numbered, then put into a sterile cloth bag covered with a sterile silicone oil paper bag, after which they were allowed to defecate autonomously. After all samples were collected, the nestlings were sent back to the nest box. The feces in each paper bag were collected and poured into 2-ml sterile frozen storage tubes labeled with the corresponding number. All samples were placed in liquid nitrogen tanks until they were transported to the laboratory, where they were frozen at −80°C until analysis. The adult birds were captured with traps set at the hole of the nest box, after which samples were collected and preserved in the same way as for nestlings. After all samples were collected, the adult birds were released near the nest. A total of 86 fecal samples were obtained, 53 of which were used in this study. These samples were collected from eight adult birds (AD, *n* = 8) and 45 nestlings during the pre-fledging period. We divided the entire pre-fledging period into the following six stages: the downy feather stage (DF, 1–5 days of age, *n* = 8); early acicle feather stage (EAF, 6–10 days of age, *n* = 8); later acicle feather stage (LAF, 11–15 days of age, *n* = 8); early contour feather stage (ECF, 16–20 days of age, *n* = 8); later contour feather stage (LCF, 21–25 days of age, *n* = 8); and qi feather stage (QF, more than 25 days old, *n* = 5). Additional detailed information for all samples is shown in Supplementary Table 1.

### DNA Extraction, PCR Amplification, and High-Throughput Sequencing

The total DNA was extracted from fecal samples using an E.Z.N.ATM Mag-Bind Soil DNA Kit according to the manufacturer’s instructions. A Qubit 3.0 DNA detection kit was used to quantify the genomic DNA after the first round of amplification and determine the amount of DNA to be added for the PCR reaction. PCR amplification of the V3–V4 hypervariable region of bacterial 16S rRNA genes was performed using the bacterial-specific forward primer 341F and reverse primer 805R (Alexander et al., 2012). The PCR reaction system and conditions were described in additional detail in the Supplementary Material. The PCR products were detected by agarose electrophoresis after PCR MagicPure Size Selection DNA Beads (TRANSGEN, Beijing, China) were used to purify the PCR amplicons. A Qubit 3.0 DNA detection kit was used to accurately quantify the recovered DNA, followed by subsequent mixing of PCR amplicons in equimolar ratios. Next, 10 ng of DNA was taken from each sample and used at a final molar mass of 20 pmol for sequencing. The construction of the Illumina MiSeq platform and sequencing of the amplification library were completed by Sangon Biotech (Shanghai) Co., Ltd.

### Bioinformatics Analysis

High-quality effective sequences were analyzed using the Quantitative Insights Into Microbial Ecology (QIIME, v1.8.0) (Caporaso et al., 2010), which has been widely used in the analysis of 16S rRNA gene sequences. Briefly, raw sequences with unique barcodes were assigned to respective samples. Sequences shorter than 150 bp, having average Phred scores of < 20, containing ambiguous bases, or sequences containing mononucleotide repeats of over 8 bp were regarded as low-quality sequences and removed (Chen and Jiang, 2014; Gilbert et al., 2016). Paired-end reads were assembled using FLASH (Magoc and Salzberg, 2011). Assembled sequences were trimmed of barcodes and sequencing primers. After detection and removal of chimeric sequences, the remaining trimmed and assembled sequences were clustered into operational taxonomic units (OTUs) at 97% sequence identity using UCLUST (Edgar, 2013). A representative sequence was selected from each OTU using default parameters. Representative sequences were then aligned against the Greengenes database (DeSantis et al., 2006) using the best hit BLAST method (Altschul et al., 1997) to taxonomically classify the sequences. An OTU table was then generated to record each OTU’s abundance per sample, along with the associated taxonomic classification. OTUs representing less than 0.001% of the total sequences across all samples were discarded.

### Statistical Analysis

A Venn diagram was generated to visualize the shared and unique OTUs among groups using the R package “Venn Diagram” (v2.4-6) (Chen and Boutros, 2011) based on the occurrence of OTUs across groups regardless of their relative abundance (Zaura et al., 2009). OTU-level ranked abundance curves were generated to compare the richness and evenness of OTUs among samples. The Chao1 index (richness estimate), abundance-based coverage estimator (ACE, richness estimate), Shannon’s diversity, Simpson’s diversity index, and observed OTUs were calculated using the OTU table in QIIME to determine the alpha diversities for the different growth stages of *F. tinnunculus*. Kruskal–Wallis tests (for non-normally distributed data) and one-way analysis of variance (ANOVA; for normally distributed data) were used to evaluate differences in alpha diversity values among age groups, and all *p* values were corrected via the false discovery rate (FDR) correction. Beta diversity was analyzed to investigate the microbial community structural variation of the different group samples using unweighted and weighted UniFrac distance metrics (Lozupone and Knight, 2005; Lozupone et al., 2007). The former only take presence/absence of microbial taxa into account while the latter incorporate not only presence/absence of taxa but also the relative abundance of OTUs. The significance of differentiation of microbiota structure among groups was assessed by PERMANOVA (permutational multivariate analysis of variance) using R package “vegan” (Jari Oksanen et al., 2019). Differences in the Unifrac distances for group pairwise comparisons were determined using the Student’s *t* test and the Monte Carlo permutation test with 1000 permutations. Beta diversity was then visualized with principal coordinate analysis (PCoA) (Ramette, 2007).

Inferred microbial functions were predicted using the phylogenetic investigation of communities by reconstruction of unobserved states (PICRUSt, v1.0.0) (Langille et al., 2013) software package and comparison against the Kyoto Encyclopedia of Genes and Genomes (KEGG) database (Nakaya et al., 2013) using the high-quality sequences. The relative abundances of predicted functions in each sample were calculated based on the abundance matrix obtained via PICRUSt, and significant differences in each function’s relative abundances among different groups were tested using analysis of variance (ANOVA) or Kruskal–Wallis tests (Acar and Sun, 2013). Results were considered significant at *p* < 0.05.

All analyses were performed in R 3.6.2 (R Core Team, 2019).

## Results

### Characteristics of Gut Microbiota Structures of *F. tinnunculus*

After quality processing, a total of 1,592,930 16S rRNA gene reads were obtained, with an average of 30,055 ± 1681 sequences per sample (median: 27,712), from which 9096 OTUs were identified to be from gut microbiotas of 53 *F. tinnunculus* samples. Venn diagrams were plotted to visualize the shared and unique OTUs (as approximations of bacterial species) among the adults and nestlings from four developmental stages of *F. tinnunculus*. A total of 4.62% of the OTUs were shared among the five groups, with unique OTUs largely being present in the down feather stage (DF, 6.67%), acicle feather stage (AF, 18.33%), contour feather stage (CF, 14.31%), qi feather stage (QF, 8.95%), and adult bird (AD, 9.92%) groups (Supplementary Figure 1).

**FIGURE 1:**
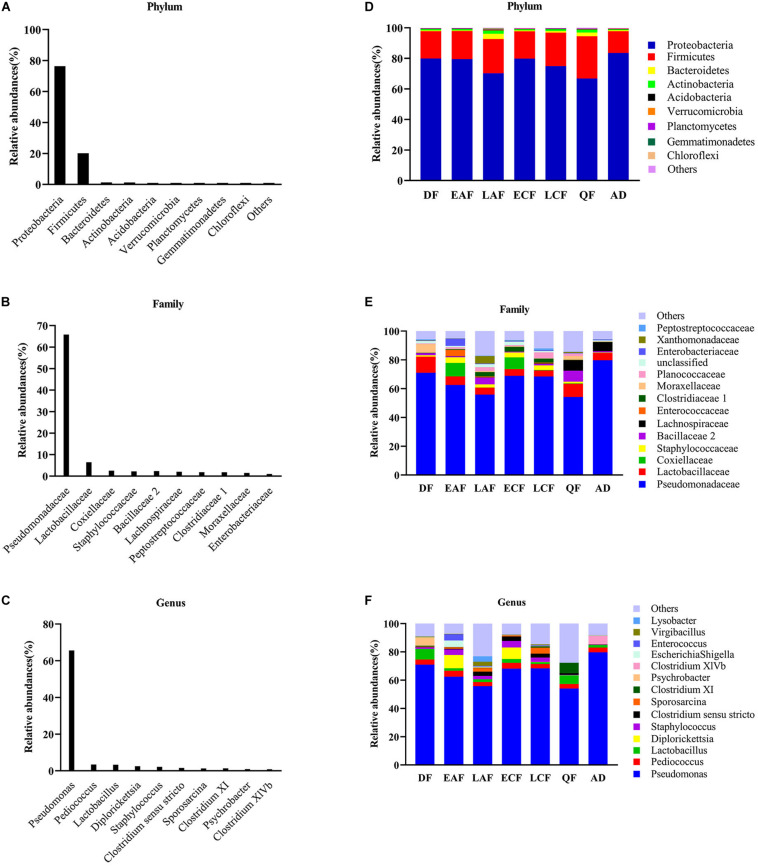
Relative contribution of the dominant phyla in all samples. **(A)** Phylum; **(B)** family; **(C)** genus. Relative abundance of these taxa in the seven sample groups. **(D)** Phylum; **(E)** family; **(F)** genus. AD refers to eight samples of adult birds; DF refers to eight samples from the downy feather stage; EAF refers to eight samples from the early acicle feather stage; LAF refers to eight samples from the later acicle feather stage; ECF refers to eight samples from the early contour feather stage; LCF refers to eight samples from the later contour feather stage; QF refers to five samples from the qi feather stage.

Nearly all reads were assignable to 45 phyla, 90 classes, 139 orders, 220 families, and 789 genera. At the phylum level, Proteobacteria were the most abundant bacterial phylum (76.38%) among *F. tinnunculus* gut communities followed by Firmicutes (20.10%), Bacteroidetes (1.30%), and Actinobacteria (1.22%) (Figures 1A,D). At the family level, Pseudomonadaceae predominated (65.82%) among *F. tinnunculus* gut communities followed by Lactobacillaceae (6.48%), Coxiellaceae (2.55%), Staphylococcaceae (2.20%), Bacillaceae 2 (2.40%), Peptostreptococcaceae (1.84%), Clostridiaceae 1 (1.79%), Moraxellaceae (1.50%), and Enterobacteriaceae (1.03%) (Figures 1B,E). At the genus level, *Pseudomonas* predominated (65.66%) the gut communities followed by *Pediococcus* (3.45%), *Lactobacillus* (3.33%), *Diplorickettsia* (2.54%), *Staphylococcus* (2.20%), *Clostridium sensu stricto* (1.60%), *Sporosarcina* (1.26%), and *Clostridium XI* (1.36%) (Figures 1C,F).

### Changes of Gut Microbiota in Different Developmental Stages of Nestlings and Differences Compared With Adults

**FIGURE 2:**
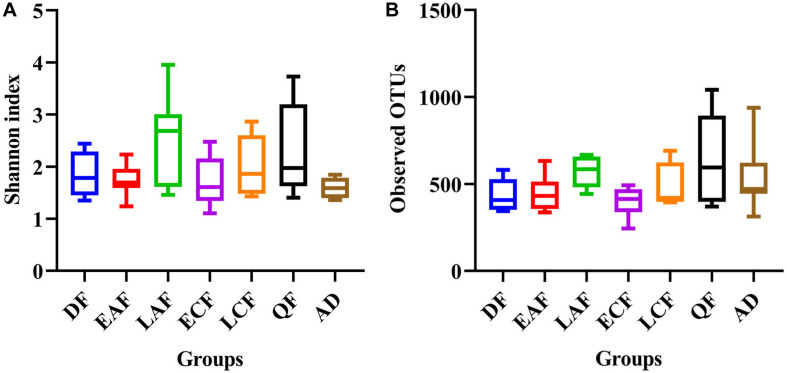
Alpha diversity of microbial communities in the *F. tinnunculus* gut. Data plotted show the means ± SE of the Shannon index **(A)** and number of observed OTUs **(B)** based on rarefaction plots (Supplementary Figure 1). AD refers to eight samples from the adult birds; DF refers to eight samples from the downy feather stage; EAF refers to eight samples from the early acicle feather stage; LAF refers to eight samples from the later acicle feather stage; ECF refers to eight samples from the early contour feather stage; LCF refers to eight samples from the later contour feather stage; QF refers to five samples from the qi feather stage.

**FIGURE 3:**
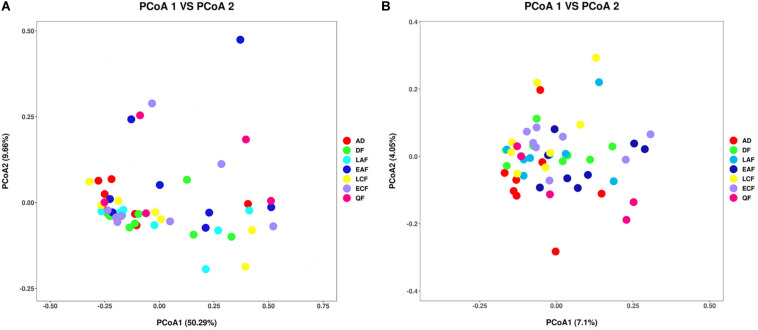
Differential gut microbiota communities across all samples. **(A)** Principal coordinate analysis plot of weighted UniFrac distances for the *F. tinnunculus* species sampled from seven developmental stages. Each point represents the gut microbiota community of an individual *F. tinnunculus*. **(B)** Principal coordinate analysis plot of unweighted UniFrac distances for the *F. tinnunculus* species sampled from seven developmental stages.

The observed OTUs, Ace, Chao1, Shannon, and Simpson indices were used to evaluate the differences in community richness and diversity among the groups. Measurements of gut community alpha diversity did not vary significantly across nestling groups at different stages of development (based on one-way ANOVA or Kruskal–Wallis tests, ACE index, *p* = 0.4181; Chao1 index, *p* = 0.3315; Shannon index, *p* = 0.0675; Simpson index, *p* = 0.194; observed OTUs, *p* = 0.3021). Thus, the gut community diversity of nestlings was similar across developmental stages (Figure 2 and Supplementary Figures 2, 3). However, there were significant differences between adults and nestlings in the gut microbiota diversity (based on one-way ANOVA or Kruskal–Wallis tests, Shannon index, *p* = 0.0101; Simpson index, *p* = 0.037). Thus, the gut microbiota diversity of *F. tinnunculus* nestlings was higher than in adults (Figure 2 and Supplementary Figures 2, 3). The differences in gut microbiota diversity among developmental stages of nestlings were also supported by patterns in β diversity. A significant effect of age was observed for microbial community membership when using unweighted UniFrac distances (Figure 3 and Supplementary Table 2; *F*_7_,_53_ = 1.11, *R*^2^ = 0.13, *p* = 0.009). However, removal of the adult samples resulted in the absence of statistical significance among groups (Figure 3 and Supplementary Tables 2, 3; *F*_7_,_53_ = 0.82, *R*^2^ = 0.12, *p* = 0.056), suggesting that the age-related structure differences were due to differences between nestlings and adults. No significant differences were observed when considering weighted UniFrac distances regardless if all data points were used or if adult samples were removed from the datasets (Figure 3 and Supplementary Tables 2, 3; *p* = 0.676 and 0.895, *F*_7_,_53_ = 1.08 and 0.65, and *R*^2^ = 0.10 and 0.08, respectively). Thus, neither microbial community membership nor structure significantly varied as an effect of developmental stage when considering only nestling developmental stages.

In addition to the differences in overall microbial diversity, different growth stages of nestlings contained an unbalanced abundance of gut microbiota groups. The relative abundance of some microbial taxa fluctuated during the development of nestlings (Supplementary Figures 4, 5). *Pseudomonas* showed a declining trend in the early stage, increased suddenly in the early contour feather stage, and then decreased. Firmicutes showed the opposite trend (Figure 1E and Supplementary Figures 4, 5). There were also differences in relative abundance of particular microbial taxa between adults and nestlings. At the phylum level, the abundance of Firmicutes (21.08%), Bacteroidetes (1.40%), and Actinomycetes (1.32%) was higher in nestlings than adult birds, while that of Proteobacteria (75.18%) was lower (Figure 1D and Supplementary Figure 6). Comparison at the family level showed that Staphylococcaceae (2.52%) and Planococcaceae (1.79%) abundance was higher in nestlings, while that of Pseudomonadaceae (79.76%) and Lachnospiraceae (6.26%) was higher in adults (Figure 1E and Supplementary Figure 6). At the genus level, the abundance of *Pseudomonas* (79.71%) and *Clostridium XlVb* (6.02%) was higher in adult bird samples, while that of *Staphylococcus* (0.25%) and *Sporosarcina* (0.02%) was lower (Figure 1F and Supplementary Figure 6). Most of the dominant genera found in chicks were different from those observed in adults. Although *Pseudomonas* showed the highest abundance in all groups, the second dominant genera varied in different developmental stages and differed from that in adults (Figure 1 and Supplementary Figure 4).

### Functional Predictions With PICRUSt

**FIGURE 4:**
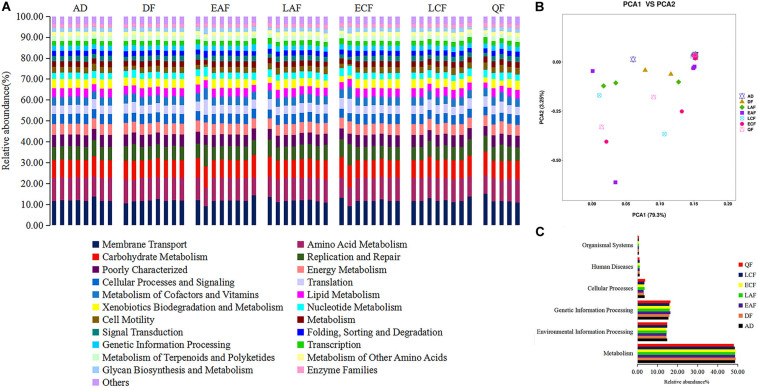
Functional predictions of all samples using PICRUSt. **(A)** Variations in functional gene families across seven groups. **(B)** Principal component analysis (PCA) showing microbial functional diversity across all samples. **(C)** Histogram showing the six categories of gene pathways across seven groups. AD refers to eight samples from adult birds; DF refers to eight samples from the downy feather stage; EAF refers to eight samples from the early acicle feather stage; LAF refers to eight samples from the later acicle feather stage; ECF refers to eight samples from the early contour feather stage; LCF refers to eight samples from the later contour feather stage; QF refers to five samples from the qi feather stage.

We used PICRUSt to predict changes in microbial functions that might be associated with changes in OTU abundance detected by 16S sequencing. The PICRUSt approach has been confirmed to be useful to predict genomes of organisms in environmental samples and may offer insights into the potential functions of *F. tinnunculus* gut microbiota. In this study, the chosen reference OTUs were used to match the KEGG database to predict microbial functions. Using this method, our study inferred 39 gene families at Level 2 in the fecal samples (Figure 4A, showing only 22 pathways accounted for more than 1%). Additionally, as shown in Figure 4B, PCA revealed that the potential functions of the microbiota of the seven groups were similar (ANOSIM, *p* > 0.05).

The majority of the 39 gene families belonged to the following six broad categories of life activity: Organismal Systems (0.76%), Human Diseases (1.14%), Cellular Processes (3.49%), Genetic Information Processing (16.14%), Environmental Information Processing (14.63%), and Metabolism (48.61%) (Figure 4C). Microbial metabolic pathways (e.g., nucleotide metabolism, carbohydrate metabolism, amino acid metabolism, and energy metabolism) comprised half of the 22 predicted most abundant pathways. Thus, the *F. tinnunculus* microbial communities likely participate in a high level of metabolic activities (Figure 4A and Supplementary Table 4).

## Discussion

There has been increased interest in the gut microbiotas of vertebrates because of their close relationships with health, growth, and development; however, few studies have investigated gut microbiota communities during the development of raptors. Raptors are carnivorous and are often regarded as pathogen vectors; studies on age-related change of gut microbiota in raptors will lay an important foundation for subsequent research on the prevalence of enteropathogens and the consequences of particular bacterial consortia in bird host health. In this study, we characterized the gut microbiota compositions of *F. tinnunculus* at various stages of development and compared the differences in gut microbiota between adult birds and nestlings in the same habitat. Consistent with the results of previous studies (Barbosa et al., 2016; Kou et al., 2019; Wang et al., 2019b), our research showed that the dominant flora of the gut microbiotas of *F. tinnunculus* were Proteobacteria, Firmicutes, Actinobacteria, and Bacteroidetes at the phylum level, indicating that the wild birds’ gastrointestinal tracts harbored a relatively conserved consortium of microbial phyla that are specifically adapted to gut conditions. As observed for other carnivorous birds (e.g., Cooper’s hawks; Taylor et al., 2019 and wintering red kites; Blanco, 2014), *F. tinnunculus* harbored a higher proportion of Proteobacteria in their gut microbiota. It is worth noting that Proteobacteria include a diversity of genera that are opportunistic pathogens including *Escherichia*, *Rickettsia*, and *Salmonella*, all of which have been isolated from *F. tinnunculus*. Given the higher relative abundances of Proteobacteria in wild bird gut microbiota, the lack of resolution regarding their *in situ* functions, and that there are many pathogens in this phylum, this group could be an important target for further research into the functions of Proteobacteria and prevalence of enteropathogens in wild bird hosts.

The gut microbiotas of birds are subject to dynamic processes as in other vertebrates, and they are regulated by numerous extrinsic and intrinsic factors (environmental influence, see van Veelen et al., 2017; location and diet influence, see Hird et al., 2014; host age influence, see Grond et al., 2017). Investigations of the age-related change in gut microbiota communities are scarce, with most so far only evaluating gut microbial communities at the end of the nestling period (Potti et al., 2002; Lucas and Heeb, 2005; Wang et al., 2019a). In this study, samples were taken every 5 days from nestlings in order to closely track changes in the gut microbiota community structure during nestling development. Unlike a previous study that demonstrated that the gut bacterial communities associated with early stages in altricial birds vary widely in diversity and abundances (Gonzalez-Braojos et al., 2012), our study showed that there were no significant differences in the diversity among all stages of nestlings, which means instead of developing gradually, development of the gut microbiota in nestlings occurs immediately after hatching. It is a challenge to find out the reasons for the non-significant change in diversity with age; our results shed new light on the dynamics of microbial diversity during the ontogeny of avian hosts and indicate that the increase in gut diversity between hatching and adulthood may not be as linear as previously suspected.

Although many studies of other species have shown that the gut microbiota changed during ontogenesis, with a significant increase in diversity (van Dongen et al., 2013; Waite and Taylor, 2014; Kohl et al., 2019), our results showed that the gut microbiota diversity of *F. tinnunculus* nestlings was higher than that of adults. Similar results were observed in precocial nestlings of arctic shorebirds (Grond et al., 2017) and Great tits (*Parus major*) (Teyssier et al., 2018). There may be several reasons for these results. First, differences in the morphological characteristics or other life history characteristics may underlie differences in microbial succession among these avian hosts (Kohl et al., 2018). Specifically, the physical and chemical properties of the gastrointestinal tracts in nestlings differ from those of adults. For example, early colonization of the gut by facultative anaerobes results in the development of an anaerobic environment over time, which then creates the anaerobic conditions required for colonization by obligate anaerobic gut microbes (Malmuthuge et al., 2015). Additionally, the immune system is believed to play a key role in formation of the gut microbiota in animals (Lavoie et al., 2007; Noreen et al., 2011). Nestlings had not developed a more adaptive immune system, which results in a large number of transient gut microbiotas at the development stage. There are many uncertain factors that lead to the temporary large-scale colonization of some species, including lack of a fully developed, relatively imperfect functional immune system of nestlings, living long term in a relatively closed nest box, and the lack of self-predatory abilities. We observed differences in the relative abundance of some gut microbiota taxa between *F. tinnunculus* adults and nestlings, with Firmicutes (21.8%) and Actinomycetes (1.23%) being higher in nestlings than in adults. Based on studies showing that the abundance of Firmicutes are associated with obesity in humans, and the existence of weight gain in chickens and rodents (Angelakis and Raoult, 2010; Clemente et al., 2012; John and Mullin, 2016), the relatively higher abundance of Firmicutes in *F. tinnunculus* nestlings may contribute to the increase of fat accumulation, thus improving the survival rate of nestlings. Functional predictions of the *F. tinnunculus* gut microbial communities further support the assertion that their gut microbial communities are mostly involved in metabolism and regulation of host body metabolisms. However, caution should be used when interpreting predictive results of PICRUSt because of its inherent limitations.

It should be noted that this study has several important limitations. First, we characterized only fecal-derived populations. It is known that different regions of the gut harbor distinct microbial communities that are adapted to local conditions such as tissue structure, host secretions, pH, and oxygen concentration. We also did not collect gut microbiotas from sub adults in the post-fledging stage, which may have resulted in the transition of gut microbiotas from nestlings to adults being missed. In addition, we did not correlate the changes in gut microbiota loads during the pre-fledging period with nestling growth and survival in the nest. We recommend that future studies of age-specific intestinal microbial communities investigate the correlation of gut microbiota loads with nestlings’ growth.

## Conclusion

In summary, this study examined the composition and diversity of the intestinal microbial communities of *F. tinnunculus*. Specifically, dynamic changes in the gut microbiota of nestlings were monitored during different growth stages, and the gut microbiota communities of nestlings and adults were compared. The results presented herein provide a theoretical reference to provide a more accurate understanding of the gut microbiota diversity of wild birds and the process of gut microbiota changes with the development of nestlings. Given the potentially profound impact of the gut microbiota on host condition, further studies of gut microbiota communities in raptors are warranted.

## Data Availability Statement

The datasets generated for this study can be accessed from NCBI Sequence Read Archive (SRA), SRR12390638 and SRR12390637.

## Ethics Statement

This study conformed to the guidelines for the care and use of experimental animals established by the Ministry of Science and Technology of the People’s Republic of China (Approval number: 2006-398). All studies were approved by the Laboratory Animal Welfare and Ethics Committee of Jilin Agricultural University. All samples were obtained without harming the study animal.

## Author Contributions

All authors listed have made substantial, direct and intellectual contribution to the work, and approved it for publication. All authors contributed to the article and approved the submitted version.

## Conflict of Interest

The authors declare that the research was conducted in the absence of any commercial or financial relationships that could be construed as a potential conflict of interest.
