# Supplementary material for: Comparative Analysis of the Gut Microbial Communities of the Eurasian Kestrel (Falco tinnunculus) at Different Developmental Stages
Source: Front Microbiol. 2020 Dec 18;11:592539. doi: 10.3389/fmicb.2020.592539 (PMC7775371; doi:10.3389/fmicb.2020.592539)
Supplement: Supplementary file 1 [file Data_Sheet_1.PDF]

Supplementary Material

Part 1 | Supplementary Figures and Tables

Supplementary Figures

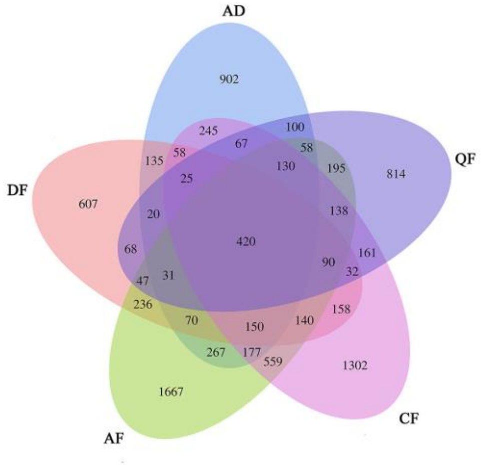

Supplementary Figure 1 | Venn diagram of shared and unique OTUs in the fecal bacterial communities among *F. tinnunculus* individuals of different developmental stages. AD, adult birds; DF, downy feather stage; AF, acicle feather stage; CF, contour feather stage; QF, qi feather stage individuals.

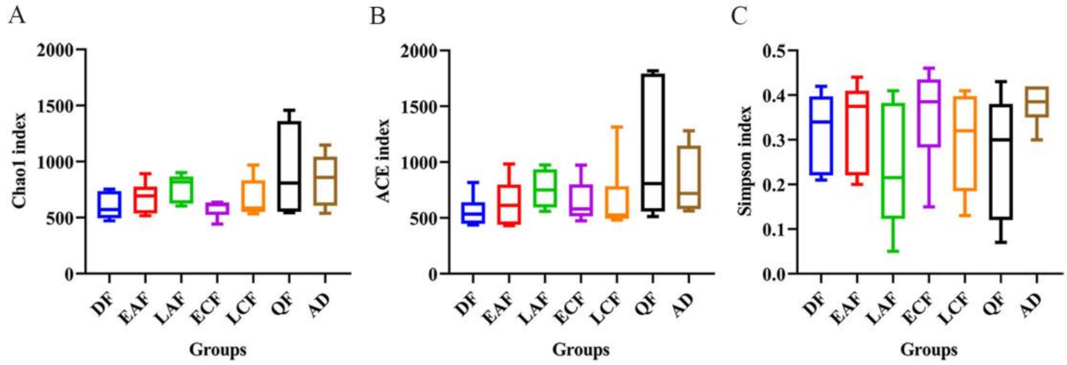

Supplementary Figure 2 | Alpha diversity of microbial communities in the *F. tinnunculus* gut. Data show the means ± SE of the Chao1 index (**A**), ACE index (**B**), and Simpson index (**C**) metrics based on rarefaction plots. AD refers to eight samples from the adult birds; DF refers to eight samples from the downy feather stage; EAF refers to eight samples from the early acicle feather stage; LAF refers to eight samples from the later acicle feather stage; ECF refers to eight samples from the early contour feather stage; LCF refers to eight samples from the later contour feather stage; QF refers to five samples from the qi feather stage.

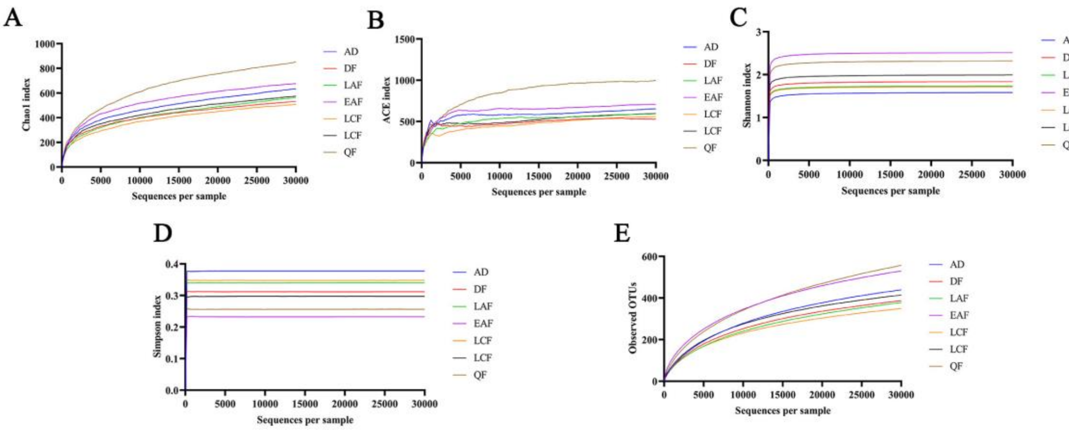

Supplementary Figure 3 | Rarefaction curves of gut microbial communities. Rarefaction analysis involved random sampling with replacement and estimation of the total number of OTUs present in the samples. The curves reach asymptote when observed diversity or richness is saturated based on the number of sequences that are sub-sampled. (**A**) Chao1 index, (**B**) ACE index. (**C**) Shannon index, (**D**) Simpson index, (**E**) observed OTUs. Symbols: AD, adult bird; DF, downy feather stage; EAF, early acicle feather stage; LAF, later acicle feather stage; ECF, early contour feather stage; LCF, later contour feather stage; QF, qi feather stage.

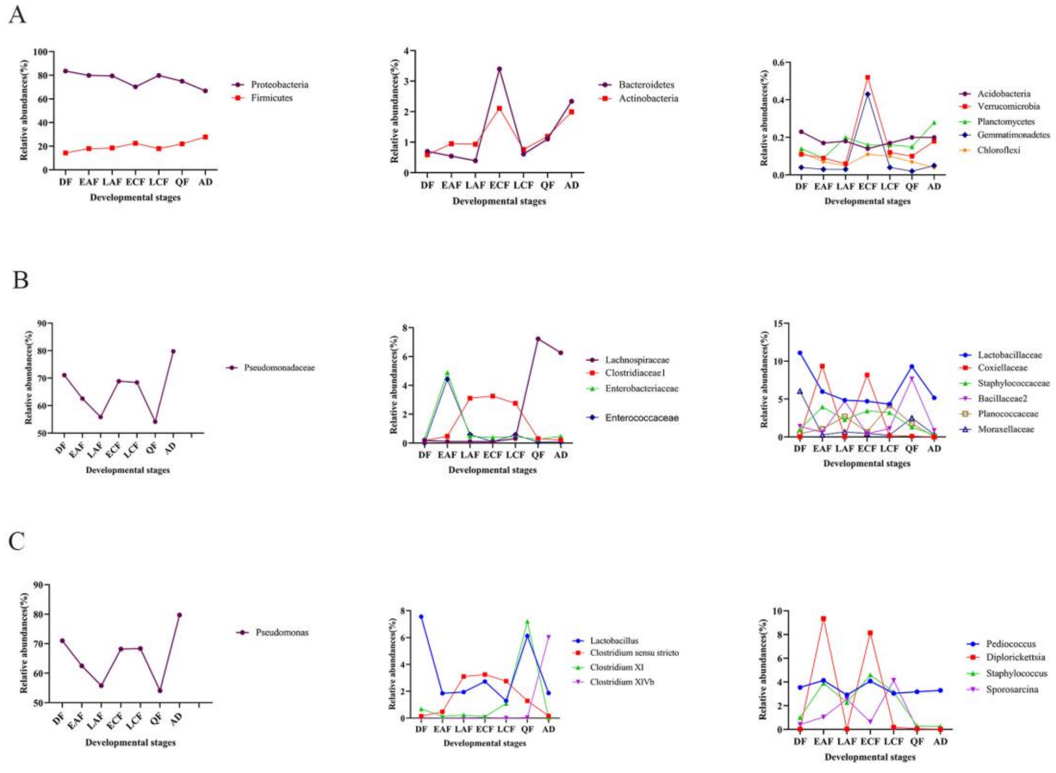

Supplementary Figure 4 | Classification of bacterial genera based on their variation

trends in the seven F. tinnunculus groups at the (**A**) phylum; (**B**) family; and (**C**) genus. AD refers to eight samples of adult birds; DF refers to eight samples from the downy feather stage; EAF refers to eight samples from the early acicle feather stage; LAF refers to eight samples from the later acicle feather stage; ECF refers to eight samples from the early contour feather stage; LCF refers to eight samples from the later contour feather stage; QF refers to five samples from the qi feather stage.

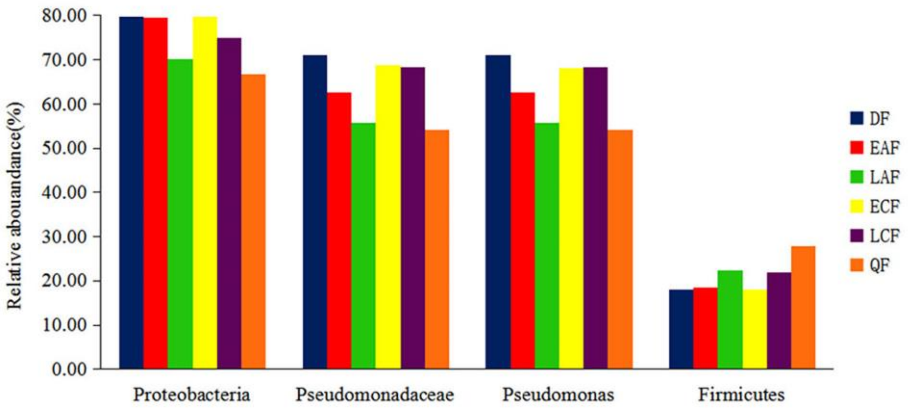

Supplementary Figure 5 | Comparison of abundance of dominant species in different developmental stages of nestlings. Symbols: AD, adult bird; DF, downy feather stage; EAF, early acicle feather stage; LAF, later acicle feather stage; ECF, early contour feather stage; LCF, later contour feather stage; QF, qi feather stage.

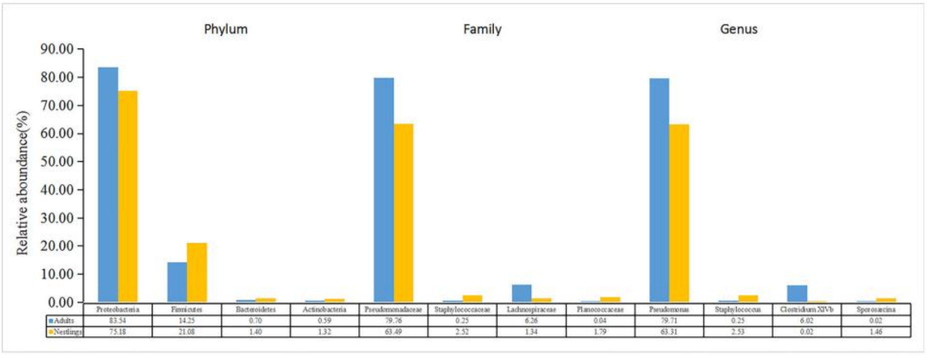

Supplementary Figure 6 | Comparison of species abundance between adults and nestlings.

Supplementary Tables

Supplementary Table 1 | Summary of sample collection information for the study F. tinnunculus

Parameter	AD (*n*=8)	DF (*n*=8)	AF (*n*=16)		CF (*n*=16)		QF (*n*=5)
			EAF (*n*=8)	LAF (*n*=)	ECF (*n*=8)	LCF (*n*=8)	
Body weight (g)	234.93±25.91a	43.37±36.25e	110.3±21.21d	157.96±33.18c	200.72±29.92b	199.34±29.84b	231.66±24.99ab
Body length (mm)	330.83±21.78a	88.63±24.13e	120.81±13.28e	171.24±23.6d	192.74±22.96cd	205.23±62.83c	250.65±13.94b
Wings (mm)	259.61±11.52a	25.08±12.85h	49.42±11.14g	81.19±13.45f	123.20±14.79e	144.09±30.95d	168.06±13.97b
Tail length (mm)	186.88±16.48a	7.37±5.59e	15.11±4.10e	45.95±12.31d	84.42±19.85c	106.77±31.71b	120.38±7.75b
Middle finger (mm)	27.14±2.63a	11.87±4.83d	18.48±4.17c	23.24±2.62b	26.02±2.83ab	27.89±3.47a	28.15±4.07a
Tarsometatarsus (mm)	42.61±2.97a	18.17±6.09d	30.54±4.03c	36.27±4.39b	46.01±2.94a	45.05±2.50a	44.39±4.89a
Claw (mm)	11.03±0.47a	4.17±1.45e	6.29±0.77d	8.36±0.61c	9.92±0.90b	10.23±0.92ab	10.35±0.89ab
Beak (mm)	15.39±2.07a	8.22±1.37d	9.78±0.42c	10.92±0.93c	12.20±1.06bc	12.70±1.14b	13.39±0.96b
Cerated beak (mm)	18.59±1.67ab	10.88±1.44e	13.35±1.37d	14.99±1.26c	15.70±1.02c	16.89±1.39bc	17.47±1.35b

Values shown are the means ± SE. Different letters indicate significant differences between sample periods ($P \leqslant 0.05$). Sample sizes (n) are in parenthes.

Supplementary Table 2 | Summary of statistical results for testing drivers of microbiome structure. Community membership utilizes unweighted UniFrac distances (which investigate the presence and absence of bacterial lineages). Community structure uses weighted UniFrac distances (which take relative abundances of bacterial lineages into account).

	unweighted UniFrac		weighted UniFrac	
	R^2	P	R^2	P
Nestlings	0.12	0.056	0.08	0.895
All Birds	0.13	0.009	0.10	0.676

$P \leqslant 0.05$ indicates a significant difference.

Supplementary Table 3 | Results of PERMANOVA analysis.

	AD vs DF	AD vs EAF	AD vs LAF	AD vs ECF	AD vs LCF	AD vs QF	DF vs EAF	DF vs LAF	DF vs ECF	DF vs LCF	DF vs QF
Phylum	0.042	0.070	0.060	0.064	0.114	0.362	0.563	0.805	0.637	0.523	0.545
Family	0.024	0.039	0.012	0.042	0.092	0.127	0.161	0.776	0.240	0.245	0.777
Genus	0.029	0.054	0.018	0.036	0.099	0.158	0.118	0.681	0.172	0.136	0.578

	EAF vs LAF	EAF vs ECF	EAF vs LCF	EAF vs QF	LAF vs ECF	LAF vs LCF	LAF vs QF	ECF vs LCF	ECF vs QF	LCF vs QF	Between
Phylum	0.582	0.806	0.687	0.895	0.807	0.766	0.752	0.758	0.971	0.957	0.514
Family	0.222	0.966	0.533	0.881	0.315	0.169	0.751	0.294	0.865	0.673	0.133
Genus	0.147	0.977	0.616	0.887	0.223	0.145	0.604	0.425	0.890	0.793	0.113

$P \leqslant 0.05$ indicates a significant difference.

Supplementary Table 4 | PICRUSt analysis showing predicted relative abundance of all KEGG pathways (Level-2 KOs)

KEGG pathways	AD(n=8)	DF(n=8)	AF(n=16)		CF(n=16)		QF(n=5)
			EAF(n=8)	LAF(n=8)	ECF(n=8)	LCF(n=8)	
Membrane Transport	12.13±0.72a	11.81±0.56a	11.92±1.43a	11.97±0.83a	11.73±1.1a	12.16±0.86a	12.24±1.66a
Amino Acid Metabolism	10.87±0.47a	10.87±0.41a	10.34±1.07a	10.71±0.48a	10.56±0.82a	10.68±0.56a	10.37±0.92a
Carbohydrate Metabolism	8.55±0.41a	8.65±0.31a	9.11±0.97a	8.93±0.45a	8.89±0.86a	8.88±0.66a	9.24±1.17a
Replication and Repair	6.57±0.49a	6.78±0.52a	7.06±1.31a	6.90±0.55a	7.11±1.38a	6.79±0.57a	7.01±0.70a
Poorly Characterized	5.62±0.16a	5.60±0.11a	5.56±0.3ab	5.50±0.2ab	5.53±0.28ab	5.56±0.19ab	5.32±0.43b
Energy Metabolism	5.21±0.03a	5.26±0.14a	5.28±0.32a	5.21±0.08a	5.31±0.29a	5.20±0.08a	5.36±0.34a
Cellular Processes and Signaling	4.68±0.18a	4.62±0.19a	4.56±0.37a	4.55±0.16a	4.55±0.38a	4.60±0.20a	4.45±0.31a
Translation	4.21±0.29a	4.42±0.40a	4.61±1.06a	4.37±0.32a	4.63±1.06a	4.34±0.36a	4.58±0.61a
Metabolism of Cofactors and Vitamins	3.94±0.10a	4.01±0.12a	4.16±0.55a	4.03±0.06a	4.20±0.54a	4.01±0.05a	4.05±0.19a
Lipid Metabolism	4.09±0.29a	4.10±0.17a	3.99±0.35ab	4.00±0.21ab	4.05±0.28ab	3.99±0.31ab	3.68±0.70b
Xenobiotics Biodegradation and Metabolism	4.24±0.53a	4.18±0.27a	3.77±1.05a	3.93±0.57a	3.88±1.00a	4.02±0.62a	3.60±1.04a
Nucleotide Metabolism	3.04±0.18a	3.19±0.32a	3.31±0.54a	3.20±0.25a	3.29±0.59a	3.16±0.29a	3.31±0.42a
Cell Motility	2.84±0.23ab	2.56±0.34b	2.40±0.55b	2.88±0.34ab	2.45±0.69b	2.79±0.25ab	3.14±0.45a
Metabolism	2.70±0.08a	2.68±0.10a	2.70±0.28a	2.64±0.10a	2.64±0.23a	2.70±0.08a	2.74±0.13a
Signal Transduction	2.44±0.06a	2.48±0.08a	2.52±0.28a	2.40±0.08a	2.53±0.26a	2.41±0.09a	2.40±0.15a
Folding, Sorting and Degradation	2.59±0.15a	2.46±0.25a	2.41±0.49a	2.45±0.22a	2.39±0.53a	2.50±0.25a	2.40±0.45a
Genetic Information Processing	2.40±0.06a	2.49±0.12a	2.53±0.27a	2.40±0.04a	2.43±0.06a	2.41±0.06a	2.48±0.12a
Transcription	2.30±0.16a	2.29±0.14a	2.31±0.22a	2.41±0.23a	2.30±0.22a	2.40±0.25a	2.46±0.37a
Metabolism of Terpenoids and Polyketides	2.25±0.16a	2.29±0.09a	2.16±0.25a	2.18±0.17a	2.20±0.20a	2.21±0.17a	2.08±0.33a
Metabolism of Other Amino Acids	2.03±0.13a	2.02±0.06a	1.93±0.20a	2.00±0.13a	1.96±0.20a	1.99±0.17a	1.88±0.30a
Glycan Biosynthesis and Metabolism	1.99±0.02ab	1.96±0.06ab	2.15±0.44a	1.90±0.22ab	2.08±0.41ab	1.88±0.2ab	1.83±0.27b
Enzyme Families	1.73±0.07a	1.75±0.07a	1.77±0.11a	1.83±0.15a	1.79±0.16a	1.79±0.14a	1.86±0.28a

Values shown are the means ± SE. Different letters indicate significant differences between sample periods ($P \leqslant 0.05$). Sample sizes (n) are in parentheses.

Part 2 | MATERIALS AND METHODS

(I) The First Round of Amplification

A Qubit3.0 DNA detection kit was used to accurately quantify genomic DNA and determine the amount of DNA that should be added to PCR reactions. The primers used for PCR were fusion primers commonly used for sequencing 16S rRNA gene V3-V4 hypervariable region amplicons on the Miseq sequencing platform.

341F primer: CCCTACACGACGCTCTTCCGATCTG (barcode)
CCTACGGGNGGCWGCAG

805R primer: GACTGGAGTTCCTTGGCACCCGAGAATTCCA
GACTACHVGGGTATCTAATCC

PCR mixtures included:

2×Taq master Mix		15 μl
F Barcode-PCR primer (10 μM)		1 μl
R Primer (10 μM)		1 μl
Genomic DNA		10-20 ng
H_20	up to	30 μl

Conditions for PCR amplification were as follows:

94℃	3min	Initial denaturation	
94℃	30s	Denaturation	5 cycles
45℃	20s	Annealing	
65℃	30s	Extension	
94℃	20s	Denaturation	20 cycles
55℃	20s	Annealing	

72℃ 30s Extension

72℃ 5min Final extension

10℃ … Recovered

(II) In the second round of amplification, Illumina bridge PCR compatible primers were ligated to the amplicons.

The PCR mixtures for the second amplification included:

2×Taq master Mix		15 μl
F Primer (10 μM)		1 μl
R Primer (10 μM)		1 μl
PCR products (previous round)		20 ng
H_20	up to	30 μl

Conditions for PCR amplification:

94℃ 3min Initial denaturation

94℃ 20s Denaturation

55℃ 20s Annealing } 5 cycles

72℃ 30s Extension

72℃ 5min Final extension

10℃ … Recovered
